# Follow-up after radiological intervention in oncology: ECIO-ESOI evidence and consensus-based recommendations for clinical practice

**DOI:** 10.1186/s13244-020-00884-5

**Published:** 2020-07-16

**Authors:** Monique Maas, Regina Beets-Tan, Jean-Yves Gaubert, Fernando Gomez Munoz, Paul Habert, Lisa G. Klompenhouwer, Paulo Vilares Morgado, Niklaus Schaefer, Francois H. Cornelis, Stephen B. Solomon, Denise van der Reijd, Jose Ignacio Bilbao

**Affiliations:** 1 Dept of Radiology, The Netherlands Cancer Institute, Amsterdam, The Netherlands; 2 Dept of Radiology, CHU Hospital Timone, Marseille, France; 3 Aix Marseille Univ, LIIE, Marseille, France; 4 Dept of Radiology, Hospital Clinic de Barcelona, Barcelona, Spain; 5 Dept of Radiology, S. João University Hospital, Porto, Portugal; 6 Dept of Radiology, Lausanne University Hospital, Lausanne, Switzerland; 7 Dept of Radiology, Memorial Sloan Kettering Cancer Center, New York, USA; 8 Dept of Radiology, University Clinic of Navarra, Calle Benjamín de Tudela, 2, 31008 Pamplona, Navarra Spain

**Keywords:** Recommendations, Interventional radiology, Oncology, Liver, Lung cancer

## Abstract

Interventional radiology plays an important and increasing role in cancer treatment. Follow-up is important to be able to assess treatment success and detect locoregional and distant recurrence and recommendations for follow-up are needed. At ECIO 2018, a joint ECIO-ESOI session was organized to establish follow-up recommendations for oncologic intervention in liver, renal, and lung cancer. Treatments included thermal ablation, TACE, and TARE. In total five topics were evaluated: ablation in colorectal liver metastases (CRLM), TARE in CRLM, TACE and TARE in HCC, ablation in renal cancer, and ablation in lung cancer. Evaluated modalities were FDG-PET-CT, CT, MRI, and (contrast-enhanced) ultrasound. Prior to the session, five experts were selected and performed a systematic review and presented statements, which were voted on in a telephone conference prior to the meeting by all panelists. These statements were presented and discussed at the ECIO-ESOI session at ECIO 2018. This paper presents the recommendations that followed from these initiatives. Based on expert opinions and the available evidence, follow-up schedules were proposed for liver cancer, renal cancer, and lung cancer. FDG-PET-CT, CT, and MRI are the recommended modalities, but one should beware of false-positive signs of residual tumor or recurrence due to inflammation early after the intervention. There is a need for prospective preferably multicenter studies to validate new techniques and new response criteria. This paper presents recommendations that can be used in clinical practice to perform the follow-up of patients with liver, lung, and renal cancer who were treated with interventional locoregional therapies.

## Key points

- Standardized follow-up after an oncologic intervention is needed to improve the quality of care.
- MRI, CT, and PET-CT are the main modalities.
- PET-CT is most valuable in lung cancer and colorectal liver metastases.
- RECIST 1.1 is suboptimal for follow-up after oncologic intervention.
- Research on adequate response evaluation methods is needed.

## Background

Interventional radiology plays an increasingly important role in the treatment of cancer. Interventional radiological treatments are mostly applied in liver, lung, and renal cancer. The main aims of follow-up after oncologic intervention are [1] early detection of residual tumor, [2] detection of local tumor progression, and [3] detection of new lesions inside the target organ or distant metastases. Another important aim is to identify complications of the intervention. In general, response to local treatment is regarded as a surrogate endpoint for long-term outcome. Currently, no evidence and/or expert-based guidelines (regarding imaging modalities and follow-up schedule) exist for follow-up after several types of radiological intervention in oncology. Therefore, in current clinical practice, follow-up is heterogeneous and consensus on the most suitable follow-up modality and schedule is lacking.

A joint session from ECIO European Conference on Interventional Oncology (ECIO) and ESOI (European Society of Oncologic Imaging), which could be attended by visitors of the conference, was held in April 2018 with expert panelists and members of the ECIO and ESOI. The aim of this session was to construct recommendations on follow-up after specific radiological locoregional interventions: ablation in CRLM, TARE in liver cancer (hepatocellular carcinoma (HCC) and colorectal liver metastases (CRLM), TACE for HCC, ablation in renal cancer, and ablation in lung cancer (primary tumors and metastases). These recommendations were based on literature and expert opinion, which were discussed among a panel of experts in both diagnostics (ESOI) and interventional oncological radiology (ECIO).

This paper reports the recommendations that follow from this joint ECIO-ESOI session and provides a guide to use in clinical practice when dealing with follow-up after locoregional interventional treatment of liver, lung, and renal cancer.

## Methods

Before the ECIO conference, experts per topic (total panelists: 5) were identified and asked to participate in the ECIO session as a panelist. A telephone conference was used to discuss the aims of the session and to start the construction of the recommendations. All participants were asked to provide a systematic review on their topic, according to the PRISMA guidelines for systematic reviews [1] for which a template was provided to the participants. The systematic review the panelists provided was a manuscript including the search question, search strategy (including in/exclusion criteria, number of hits, databases used), selection strategy of the identified papers, list of references), and a summary and discussion of the results from the included papers. Expert panelists were allowed to invoke assistance from colleagues for this systematic review.

The following topics were addressed:

1. Follow-up after radiological intervention for colorectal liver metastases, comprising of radiofrequency ablation (RFA), microwave ablation (MWA) (M.M., diagnostic radiologist).
2. Follow-up after transarterial radioembolization (TARE) for CRLM (N.S., diagnostic radiologist).
3. Follow-up after radiological intervention for hepatocellular carcinoma (HCC) by transarterial chemoembolization and TARE (P.V.M. and N.S., interventional radiologist and diagnostic radiologist).
4. Follow-up after radiological intervention for renal cell carcinoma (RCC) by RFA or MWA (S.S., interventional radiologist).
5. Follow-up after radiological intervention for primary lung cancer and lung metastases by RFA, MWA, and cryoablation (J.G., interventional radiologist).

The purpose of the search for all topics was the evaluation of accuracy or efficacy of imaging modalities to detect local tumor persistence, progression, or tumor recurrence. General exclusion criteria were [1] case reports, [2] meta-analysis, [3] reviews, [4] other locoregional therapies than specified above, [5] other language than English, and [6] mixed cohort studies where data on specific disease separately was not derivable. During the search, initially, intrahepatic cholangiocarcinoma was included but was later excluded given the lack of evidence on this topic. The details on the search question and strategy per topic are specified in Table 1.

**Table 1:** Search details

Topic	Disease	Intervention	Modalities	Hits	Articles included
Thermal ablation in CRLM	Colorectal liver metastasis	Thermal ablation techniques: radiofrequency, microwave	CT, MRI, FDG-PET(-CT), CE-US	497	14
TARE in liver cancer	Liver metastasis, intrahepatic cholangiocarcinoma, hepatocellular carcinoma	Transarterial radioembolisation (Yttrium90/TARE/SIRT)	CT, MRI, FDG-PET(-CT), Y90 SPECT/CT/PET	128	12
TACE in HCC	Hepatocellular carcinoma	Transarterial chemoembolization	CT, MRI, FDG-PET(-CT), CE-US	417	69
Thermal ablation in RCC	Renal cell carcinoma	Thermal ablation techniques: cryoablation, radiofrequency, microwave	CT, MRI	518	39
Thermal ablation in NSCLC	Non-small cell lung cancer	Thermal ablation techniques: radiofrequency, microwave	CT, MRI, FDG-PET(-CT)	181	21
Exclusion criteria all topics	Case reports, meta-analysis, reviews, other locoregional therapies, other language than English, mixed cohort studies where data on specific disease separately was not derivable.				
Purpose in all topics	Evaluation of accuracy or efficacy of imaging modalities to detect local tumor progression or tumor recurrence.				

*Abbreviations*: *CRLM* colorectal liver metastases, *HCC* hepatocellular carcinoma, *RCC* renal cell carcinoma, *NSCLC* non-small cell lung cancer, *CT* computed tomography, *MRI* magnetic resonance imaging, *FDG-PET* fluorodeoxyglucose positron emission tomography, *CE-US* contrast-enhanced ultrasound, *SPECT* single-photon emission computed tomography, *TARE* transarterial radioembolization, *SIRT* selective internal radiation therapy, *TACE* transarterial chemoembolization, *Y90* Yttrium90

After receipt of all the systematic reviews a second telephone conference was held to establish consensus on the conclusions and to summarize statements and recommendations derived from the systematic reviews as a basis for the session at ECIO 2018. All panelists approved the final statements and recommendations for each topic. The telephone conferences and establishment of consensus were moderated by R.B.T., J.I.B., and M.M..

The statements were sent to all panelists and they were asked to vote. All statements on which ≥ 80% agreement was reached were accepted as recommendations. All statements with < 80% consensus were discussed in a telephone conference and statements were deleted or adapted to achieve ≥ 80% consensus.

The result of the systematic reviews and telephone conferences with the experts are the bases of the currently presented recommendations. Final recommendations were based on a combination of systematic reviews, consensus meetings, and expert opinions. In case of limited evidence, expert opinion was the basis for recommendations.

## Results

For follow-up after the oncologic intervention, the preferred modalities were found to be: CT, PET-CT, and MRI. In some cases, ultrasound (sometimes contrast-enhanced) is also an option. Proposed follow-up schedules and imaging protocols (for liver, kidney, and lung) are presented in Table 2. The results per topic are presented below. Table 3 presents the imaging features per topic that have been identified to indicate local tumor progression (LTP). The statements that were voted and the items that were discussed after voting are provided in supplementary material S1. Also, in the supplementary material, the consensus statements and recommendations per topic can be found (S2).

**Table 2:** Recommended imaging protocol and follow-up schedule

	Kidney	Liver	Lung
Follow-up schedule	First year: 1, 3, 6 , 12 months Thereafter: every 12 months (chest imaging every 12 months)	First year: 1, 3, 6, 9, 12 months Thereafter: every 6 months Consider shortening of interval in high risk patients or other signs of recurrence	First year: 1, 3, 6, 9, 12 months (optional: before discharge after ablation) Thereafter: every 6 months
Recommended modalities	1. CT or MRI of the kidneys 2. Biopsy only after 6 months in case of suspected recurrence	1. Primary MRI (mandatory after TACE with Lipiodol), secondary CT 2. PET-CT only for metastases > 2cm and not for HCC 3. PET-CT (if available) within 48h after ablation 4. PET-CT as a problem solver and in case of suspected LTP 5. Biopsy of ablation margin can be considered in margins < 5mm (➔optional to replace MRI at 6 and 12 months by PET-CT)	1. CT 2. PET-CT not before 6 months, only in case of suspected recurrence (and targeted biopsy for recurrence), whole-body evaluation
Computed tomography	unenhanced	unenhanced (HCC)	unenhanced CT optional to establish enhancement compared to baseline late arterial (35s) after treatment
	arterial phase (20-30s)	late arterial (35-40s) (HCC)	
	nephrogenic phase (100s)	portal-venous (70s) (CRLM+HCC)	venous (70s) optional, if available
	delayed phase (10 min; to assess complications)	late venous (≥120s) (HCC)	
	slice thickness maximally 3 mm	slice thickness maximally 3 mm	slice thickness 1 mm
Magnetic resonance imaging	axial T2W (with and without fatsat)	axial GRE in and opposed phase T1W	
	axial and coronal dual echo	axial T2W FSE (with and without fatsat	
	axial dynamic 3D GRE before and after Gd (20/70/180s)	axial dynamic 3D fatsat GRE before and after Gd (20/70/180s)	
	axial 5 min post contrast GRE	axial 20 min after hepatobiliary specific Gd agent	
	axial DWI	axial DWI	
		subtraction images are highly recommended for HCC	
		axial and coronal T2 HASTE	
PET-CT	NA	Only in CRLM: standard protocol for FDG-PET-CT	standard protocol for FDG-PET-CT

*Abbreviations: CT* computed tomography, *MRI* magnetic resonance imaging, *HCC* hepatocellular carcinoma, transarterial chemoembolization, (*FDG-)PET* (fluorodeoxyglucose) positron emission tomography, *LTP* local tumor progression, *CRLM* colorectal liver metastases, *GRE* gradient echo, *FSE* fast spin echo, *fat sat* fat saturation, *Gd* gadolinium, *DWI* diffusion-weighted imaging, *HASTE* half-fourier acquisition single-shot turbo spin-echo

**Table 3:** Imaging features during follow-up per intervention

	Thermal ablation for CRLM	TARE for CRLM	TACE&TARE for HCC	Thermal ablation for renal cancer	Thermal ablation for lung cancer
CT	* increase of ablation zone size * small size difference between metastasis and ablation zone * ablation rim discontinuity or irregular shape	* Choi outperforms RECIST 1.1 for response assessment * arterial perfusion has potential to assess response (reduction in arterial perfusion predicts outcome in liver mets, not HCC)	* residual arterial enhancement (thick, nodular or irregular ring, especially with wash-out)	* early peripheral enhancing rim that sustains after 3 months * focal or nodular enhancement at ablation margin (enhanced > 15 HU at CT * increase in scar volume after 2 months post-ablation	* increase in size of the ablation scar * appearance of nodular, irregular, eccentric solid component in or at the margin of ablation zone * new contrast enhancement > 15 HU
MRI	* increase of ablation zone size * small size difference between metastasis and ablation zon same as on CT * T2 moderate high signal (focal, eccentric or nodular) at the margin * persistent high signal intensity on high b-value DWI and low ADC * disruption of the interface between ablation zone and liver parenchyma * T1 hyperintensity > 9 months * thickened or irregular peri-ablation zone tissue rim (low T1 & high T2)	* DWI at 4 weeks can predict outcome and potentially can outperform PET-CT * caveat false positives due to inflammation	* same as for CT * DWI and DCE seem promising	* same criteria as for CT * enhancement: >15% increase in signal intensity on MRI is significant) * after cryoablation: interrupted T2 low intensity rim	NA
PET-CT	* focal, eccentric or rim shaped FDG-uptake after 4-6 months post-ablation	* reduction of ≥50% in SUVmax (FDG-PET-CT) at 4 weeks predicts outcome	NA	NA	* new FDG uptake in the scar > 6 months after ablation

*Abbreviations: CRLM* colorectal liver metastases, *TACE* transarterial chemoembolization, *HCC* hepatocellular carcinoma, *TARE* transarterial radioembolization, *SIRT* selective internal radiation therapy,*CT* computed tomography, *RECIST* response evaluation criteria in solid tumors, *HU* hounsfield units, *MRI* magnetic resonance imaging, *DWI* diffusion-weighted imaging, *ADC* apparent diffusion coefficient, *DCE* dynamic contrast-enhanced, *NA* not applicable, *PET* positron emission tomography, *FDG* fluorodesoxyglucose; *SUVmax* maximum standardized uptake value, *LTP* local tumor progression

## A. Liver cancer: thermal ablation of CRLM, TARE of CRLM, and intra-arterial therapy (TACE and TARE) of HCC

### 1. Follow-up after thermal ablation for colorectal liver metastases: RFA and MWA

Thermal ablation for colorectal liver metastasis is a potential curative option in patients who are not eligible for curative resection. Overall, survival is similar to resection, as long as maximally 5 lesions up to 3 cm are ablated [2]. The main imaging modalities for follow-up are CT, MRI, and PET-CT. Both a residual tumor and a recurrence during follow-up are defined in the term local tumor progression.

#### Computed tomography

Computed tomography is widely available and commonly used, but distinguishing normal post-ablation effects from residual disease and recurrence can be challenging. Some studies show that some CT features have the potential to indicate a high risk of local tumor progression [3–6]. An increase in the volume of the ablation zone during follow-up, > 4–6 months is highly suggestive for local tumor progression. Kele et al. showed that ablation zones without local recurrence all decreased in volume during follow-up (*N* = 58 patients and 117 lesions) [5]. Wang et al. found that the risk for local tumor progression was smaller with the increasing volume of the ablation zone (i.e., a larger margin relative to the metastasis) and when the metastasis was more centrally treated, (*N* = 73 patients and 117 lesions) [6]. This is in line with the general advice to achieve an ablation margin relative to the metastasis of at least 5 mm and preferably 10 mm [6, 7]. Morphological analysis of ablation zones on CT performed immediately after RFA might be helpful in detecting local residual tumor. Schraml et al. found that on the CT images made immediately after ablation, discontinuity of the ablation zone rim was indicative of residual tumor and local tumor progression. Additionally, irregularly shaped ablation zones had a tendency to higher regrowth rates [4]. CT perfusion was shown to be helpful in a promising but very small study by Meijerink et al. (*N* = 10): arterial hyperperfusion and portal venous hypoperfusion in the rim of the ablation zone were both associated with regrowth [8]. This approach is still highly experimental.

#### FDG-PET-CT

Several studies showed a benefit for PET-CT over CT in detecting local tumor progression after ablation [9–11]. Nielsen et al. showed that a rim-shaped FDG uptake 5 months after the ablation is predictive for local tumor progression, but this rim-shaped FDG uptake can be false-positive due to inflammation up to 4–6 months [12]. Three studies with in total of 43 patients showed that PET-CT imaging shortly after RFA can be helpful in detecting complete ablation considering images are made before inflammation effects occur. Liu et al. performed PET/CT imaging within 24 h, Khandani et al. within 41 h, and Langenhoff et al. within 3 weeks after treatment [13–15]. Early PET-CT (< 48 h after ablation, e.g., in room immediate PET-CT) might overcome the false-positive findings at PET-CT later during follow-up.

#### MRI

Only 1 study that met our inclusion criteria was found for MRI in the follow-up after RFA. Kuehl et al. observed a comparable accuracy of MRI and PET/CT to detect local tumor progression in a study of 16 patients with 20 lesions. They regarded new focal hypointense T1 lesions of focal T2 hyperintense lesions at the ablation margin as signs of LTP. Sensitivity and specificity were 73% and 100%, respectively, for MRI and 84% and 100% for PET/CT [16]. A study by Kierans et al. (who evaluated a miscellaneous group of malignant liver lesions) showed that high signal intensity on unenhanced T1 > 9 months after the procedure and well-defined enhancement was more frequently encountered in patients with LTP after RFA. For MWA, a low central signal intensity at unenhanced T1 accompanied with high T1 signal intensity at the edges was found frequently, regardless of response [17]. In a review by Sainani et al., focal, eccentric, nodular moderately hyperintense areas of T2 hyperintensity (with enhancement) were reported to be suspicious for LTP, just as disruption of the interface between ablation zone and liver parenchyma. Important pitfalls are postablation edema, apparent arteriovenous shunting, and neovascularization around the ablation zone, which are signs of inflammation and LTP [18]. Diffusion-weighted MRI has been sparsely evaluated, but Schraml et al. reported that it can be helpful in detecting LTP, but the false-positive high signal on b1000 images is often encountered and is thought to be caused by post-procedural changes around the ablation zone. Follow-up usually shows resolution of these findings if LTP is absent [19].

#### Ultrasound

Currently, there is no adequate evidence available for the use of ultrasound in the detection of local tumor progression after RFA for colorectal liver metastases [20].

#### Ablation zone biopsy

One study looked at the prognostic value of biopsy of the center and margin of the ablation zone immediately after treatment for time to local tumor progression. Viable tissue in the specimen of the biopsy was an independent risk factor for local tumor progression. In a multivariate analysis, a negative biopsy and an ablation margin of more than 5 mm predicted a recurrence risk of only 3% [7].

#### Recommendations

- MRI and PET-CT are the primary recommended modalities, CT is the secondary recommended modality
- MRI and PET-CT seem to be comparable in accuracy to detect local tumor progression, depending on the time point of follow-up
- PET-CT is superior to CT during follow-up after thermal ablation (RFA/MWA) to detect local tumor progression
- PET-CT seems most useful to detect local tumor progression shortly after ablation (24–48 h) or after 4 months
- PET-CT is only indicated for metastases > 2 cm
- MRI might suffice with PET-CT as a problem solver or in case of suspected recurrence > 6 months (mostly based on expert opinion)

o MRI protocol should consist of T2, T1, DWI, multiphasic post-contrast sequences, and delayed imaging in case Primovist is used

### 2. Follow-up after TARE for CRLM

TARE is mostly used as a treatment option in patients with (colorectal) liver metastases and HCC. Follow-up after liver-directed radioembolization of liver cancer (TARE) remains a challenge. The main problems for early response assessment are the inflammatory changes after high-dose radiation and generally delayed morphologic response to TARE on imaging. The main response assessment modalities consist of FDG-PET (CT), CT, and MRI.

#### FDG-PET-CT

A reduction in metabolic activity measured by SUVmax precedes the anatomical size reduction in metastatic colorectal cancer (CRLM) [21]. Other series in mixed histologies confirmed this finding and reported that FDG-PET detected responders 6 weeks after the intervention, while only 13% of these responders showed a reduction in size (partial response) on anatomical images [22]. More recent studies confirmed the prognostic role of early FDG-PET in CRLM after TARE. Four weeks after the intervention, a reduction of SUVmax of at least 50% predicted a difference in survival of 10 months versus 4 months in CRLM [23]. More recently, advanced response criteria (PERCIST) have been evaluated to assess response in TARE patients. A change in SUVpeak and total lesion glycolysis predicted overall survival (*p* = 0.039; hazard ratio [HR], 0.24; 95% confidence interval [CI], 0.06–0.93), progression-free survival (*p* = 0.016; HR, 0.15; 95% CI, 0.03–0.69), and time to intrahepatic progression (*p* = 0.010; HR, 0.16; 95% CI, 0.04–0.65) [24]. Interestingly, in the same study, summed baseline CT diameter of less than 8 cm for the 2 largest liver metastases predicted time to intrahepatic progression (*p* = 0.013; HR, 0.21; 95% CI, 0.06–0.72) but did not predict overall or progression-free survival [24]. Overall, the body of evidence supports that a reduction of FDG avidity in early PET (4 weeks) might be useful to predict the further outcome of the patients.

#### Computed tomography

CT relies mainly on size criteria for response assessment (RECIST 1.1), which is difficult to use as a response criterion, as it does not take into account the necrosis, cystic degeneration, and edema that occurs [25]. A recent study investigating different criteria found that RECIST 1.1 after TARE is not suitable to assess response in these patients. Alternatively, Choi criteria and difference in tumor attenuation can predict outcome after TARE in CRLM patients and has the same predictive power as the EORTC PET response criteria [26]. More recently, arterial perfusion (AP) CT was investigated to predict and prognosticate outcome in TARE patients. A study by Reiner et al. showed a reduction of AP in early CT after TARE as the predictor for outcome in patients with liver metastases. Other studies measured AP of the entire tumor volume via voxel-wise histogram analysis [27]. AP derived from histogram analysis was significantly different in responders versus non-responders. Overall, the paradigm that CT might not be an imaging of choice due to the restrictions of RECIST 1.1 has to be re-challenged in the light of new response criteria and more advanced protocols as arterial perfusion protocols. Future studies are needed to determine the most suitable imaging modality after TARE.

#### MRI

Enhancement around a treated lesion after TARE can be found on MRI and is usually found when the follow-up scan is performed early (< 3 months after TARE) and then corresponds to inflammation. It can be present in the whole treated area. It is important not to mistake this as a viable tumor or progression and take into account the time from the treatment to follow-up. Recent studies focused on predicting outcome using differences in the apparent diffusion coefficient (ADC) on diffusion-weighted imaging (DWI) [28]. A study investigated 44 CRLM patients who underwent DWI before and around 1 month after TARE. An increase of the minimal ADC (minADC) of more than 22% could independently predict the overall survival (18 vs. 5 months; *p* < 0 .001), together with hepatic tumor burden [29]. DWI-MRI before and 6 weeks after TARE showed a higher positive predictive and a higher negative predictive value for the detection of response compared to FDG-PET (96% versus 88%; 96% versus 56%). Overall, the detection of response was higher for DWI-MRI than for FDG-PET-CT (96%; 22/23 versus 65%; 15/23) (*p* < 0.02) [29]. So, DWI-MRI might outperform early FDG-PET to detect progression or predict long-term outcome.

#### Radiation-induced changes in the liver due to TARE

Y90 radiation-mediated damage is mostly related to the endothelial lesion that later provokes epithelial damage [30]. After Y90 radioembolization some of the changes in the tumor that are not related to the size changes may reflect tumoral response or progression. The appearance of hemorrhagic necrosis after the treatment can induce tumor growth that is not related to progression [31]. Furthermore, despite the fact that lack of humoral enhancement can be considered as a marker of response, its absence is not a sign indicating failure of the treatment [26]. There are also other changes that do not imply any oncological meaning and that are normal reaction to the radiation, these changes include peritumoral edema, peripheral rim enhancements (of < 5 mm thickness), poorly defined areas of hypoattenuation, volumetric changes (typically ipsilateral atrophy with contralateral hypertrophy when selective treatments), capsular retraction without cirrhosis (in metastatic non-cirrhotic livers), and perihepatic and pleural effusion [32].

#### Conclusion

Initially, early (4 weeks) FDG-PET-CT or DWI-MRI seemed to be a good method to assess response and outcome after TARE in CRLM. However, enhancement-based parameters on MRI and CT (such as Choi and possibly assessment of arterial perfusion) might be of value as a stand-alone or complementary modality. Studies are still sparse and efforts should be made to run prospective clinical trials to better assess the available modalities and harmonize response criteria and follow-up.

#### Recommendations

- FDG-PET-CT is recommended to assess early response 4 weeks after TARE in CRLM
- Size-based criteria using RECIST 1.1 are not reliable for follow-up of patients, while ceCT with the use of Choi criteria might be a suitable alternative
- Early contrast-enhancement on MRI is usually caused by inflammation and diffusion-weighted imaging can help distinguish inflammation from tumor
- Arterial perfusion on ceCT has the potential to assess response, but new studies need to confirm these findings

### 3. Follow-up after TACE or TARE for HCC

Locoregional treatment of hepatocellular carcinoma (HCC) nowadays consists mainly of intra-arterial therapies: transarterial chemoembolization (TACE) and transarterial radioembolization (TARE). Early detection of residual tumor or recurrence leads to better outcomes [33]. Because of the complexities in therapeutic approaches, a therapy-tailored imaging evaluation of HCC after therapy is of paramount importance [34]. Focus is aimed at the margins of the treated area and on the viability of the treated tissue. Currently, EASL and mRECIST criteria are recommended for response assessment in HCC and can be used on dual-phase MRI or CT (arterial and portal venous phase) [35] and take into account size and vascularity.

#### Computed tomography

CT has long been the mainstay in HCC imaging for both initial tumor characterization and post-treatment follow-up. After locoregional treatment, a central area of coagulative necrosis with transient surrounding hyperemia is often seen and both will mostly resolve during follow-up. This coagulative necrosis will result in hyperdensity on unenhanced CT. Transient hyperemia is a physiologic reaction and manifests as a thin, uniform enhancement of the treated zone. After TARE, this can be visible in the whole treated area. However, small foci of the residual tumor may be obscured by transient hyperemia. Therefore, persistent arterial enhancement with washout on delayed phase images can indicate residual tumor or recurrence. Any nodular or thick areas of arterial enhancement along the treatment margin are very suspicious for residual tumor or recurrence, especially if there is a washout. In a recent retrospective study, a lack of tumor enhancement and peripheral ring enhancement showed a low risk for progression, while peripheral nodular enhancement on arterial phase CT after TACE with drug-eluting beads was associated with a likelihood of 83% for tumor progression [36]. In inconclusive cases regarding residual tumor or recurrence, an additional follow-up after 3 months is advised.

After TACE using Lipiodol, unenhanced CT can be performed to assess Lipiodol distribution. In general, complete retention of iodized oil has a high correlation with complete lesion necrosis, while incomplete retention of Lipiodol can be related to both necrosis and residual viable tumor [35, 37]. Lack of iodized oil uptake immediately after therapy may also indicate an aberrant vascular supply to the tumor and will require repeated treatment as soon as possible. Multiphasic MDCT must still be performed to further assess the areas lacking iodized oil for enhancement and washout [38]. Necrotic nonviable tumor tissue will continue to retain the oil indefinitely but will decrease in size over time. Volumetric assessment of the treatment zone is particularly helpful in cases in which necrosis is heterogeneously distributed in HCC and mRECIST is less suitable for response assessment [39, 40]. Additionally, per voxel analysis of the enhancement of the tissue might increase accuracy for response monitoring after TACE for HCC, but this has only been suggested in a small retrospective study [41]. Perfusion CT gives the opportunity to capture CT attenuation as a function of time, which can be used to quantify tissue and tumor vascular characteristics. TACE reduces tumor blood volume, which will lead to a significant decrease in hepatic arterial fraction and perfusion in tumors effectively treated by TACE. In a recent study from Su et al. (prospective study, *n* = 39, perfusion CT performed before cTACE) found that the responders (mRECIST criteria) demonstrated higher hepatic arterial perfusion (HAP) and lower hepatic portal perfusion (HPP) compared to non-responders among the 34 lesions without portal vein tumor thrombus. So far, the evidence is limited and clinical value of arterial perfusion is yet to be established.

#### MRI

The coagulative necrosis that occurs will lead to a hyperintense signal on unenhanced T1-weighted imaging. Subtraction images can be a helpful adjunct for differentiating hemorrhage from enhancing tumor on MRI [42, 43]. MR has been shown to be advantageous in assessing treatment-related changes in HCC (mainly post-contrast T1W MRI and DWI) and is superior to ceCT in evaluating patients who have undergone Lipiodol-based TACE therapies. Lipiodol does not adversely affect MR signal intensity, while beam-hardening effects on CT may obscure small enhancing tumors [44, 45]. Therefore, MRI is recommended when lipiodol-based TACE has been performed. The added value of gadoxetic acid over extracellular GBCAs after HCC locoregional therapy is unclear. Diffusion-weighted imaging can be of help in distinguishing viable from necrotic areas after HCC treatment. Residual diffusion restriction is suggestive of residual viable tumor. Combining DWI with conventional MRI can increase sensitivity for detecting viable tumor [44]. Sensitivity for detection of (residual) HCC does not improve and one study even reported a poorer diagnostic performance compared to contrast-enhanced multiphasic T1W MRI [46]. Conflicting results have been reported regarding the value of pre-treatment ADC measurements [47, 48]. There might be a value for ADC measurements after treatment, as two studies found that an increase in ADC after treatment was associated with good response [47, 48]. There is some preliminary evidence for dynamic contrast-enhanced (DCE-MRI; in which images are continuously acquired to assess perfusion by obtaining a signal-intensity-time curve) for response assessment after TACE for HCC, but the evidence is too sparse to recommend its use [49]. Future trials will have to provide evidence of the value of DCE-MRI.

#### FDG-PET-CT

HCCs are often non-FDG-avid. FGD-PET has limited value in HCCs due to its limited sensitivity for HCC detection of around 60% [43]. However, one study published that when an HCC is FDG-avid, metabolic responders survived longer than non-responders (10 vs. 5 months, respectively) [50]. Currently, FDG-PET is not recommended in HCC.

#### Ultrasound

Ultrasound is generally used for HCC screening and is not recommended for follow-up of patients with treated HCC. Contrast-enhanced EUS (CEUS) has shown potential for the detection of small foci of residual tumor after TACE [51] Also, CEUS can be used during or immediately after locoregional therapy. Until now, no convincing evidence has been published to support the use of CEUS in clinical practice for HCC [51, 52].

#### Recommendations

- MRI is the recommended follow-up modality; CT is the next best modality
- MRI is mandatory when conventional TACE with lipiodol has been performed, multiphasic CT can be performed to assess lipiodol distribution
- CT protocol should include a quadruple-phase CT protocol including unenhanced images
- There is no (established) role for PET-CT or (contrast-enhanced) ultrasound in follow-up
- During follow-up, modified RECIST or EASL criteria should be the basis to identify recurrence
- Sufficient evidence is lacking on new techniques such as DWI/DCE-MRI and perfusion CT but these techniques seem promising

### 4. Follow-up schedules after locoregional treatment of the liver

Due to the limitations of ceCT, PET-CT, and MRI are recommended modalities to monitor the treatment zone in CRLM. Nielsen et al. suggested a 3–6 monthly follow-up scheme with PET-CT for the first year after RFA. However, they reported that PET-CT might be of minimal value in follow-up after ablation of < 2 cm lesions, as the risk for local tumor progression is very low [12]. Kuehl et al. proposed the following follow-up protocol: immediately after treatment PET-CT is more useful than MRI, at 4 weeks MRI is preferred, at 3 months MRI is comparable to PET-CT (which would make MRI the preferred modality), after 6 months and 1 year PET-CT and MRI are both valuable modalities with PET-CT having the benefits of whole-body screening [16]. Thus, a combination of PET-CT with MRI was recommended.

For HCC, the proposed surveillance schedules after liver-directed therapy include: at 1 month and every 3 months thereafter, or with the interval stretched to every 6 months after 1-year post-treatment.

Closer follow-up or immediate imaging may be indicated in case of a high risk for local recurrence if there is suspicion for incomplete treatment, equivocal imaging findings, new clinical symptoms, rising alpha-fetoprotein, or other abnormal laboratory test values. This is consistent with previous work showing that the optimal time between scans is approximately proportional to the reciprocal of the square root of the probability of recurrence. Boas et al. (*N* = 1766 consecutive TACE, TARE, and RFA procedures in 910 patients) showed that recurrence is 6.5 times more likely in the first year after treatment, compared to the second year after treatment [46]. Furthermore, they showed that more frequent follow-up screening than the minimum published recommendation is cost-effective.

#### Recommendations for follow-up schedule (Table 2):

- Imaging at 1, 3, 6, 9, 12 months in the first year and every 6 months in the subsequent years
- In patients with a high risk for recurrence (ex. infiltrative type, irregular necrosis after TACE, or poor lipiodol deposition after cTACE), some evidence exists to shorten follow-up intervals in the first year in HCC
- In case of other indications for a recurrence (e.g. increasing CEA in CRLM or equivocal findings on imaging), an additional follow-up moment can be scheduled

## B. Follow-up after thermal ablation for renal cancer

Thermal ablation for renal cancer is considered a therapeutic option if complete ablation can reliably be achieved [53]. Radiofrequency ablation (RFA), cryoablation, and microwave ablation (MWA) are options in ablation for renal cancer. The visualization of the physical changes caused by freezing whether using CT, MRI, or ultrasound seems to be a major advantage of cryoablation [54, 55]. This could be useful for monitoring the treatment when the lesion to be treated is close to sensitive organs or structures.

Both CT and MRI may be used for follow-up after renal ablation [56–59]. (FDG-)PET-CT does not play a role in renal cancer, due to the low FDG avidity of RCCs. Close follow-up is usually performed in the first year after ablation at a frequency of 3–4 examinations progressively spaced, often at 1, 3, 6, and 12 months. Then, an annual follow-up is recommended for at least 5 years but often more. Follow-up imaging at 1 month after ablation is recommended to assess for complications and set baseline for future follow-up. Imaging follow-up has to detect any complications occurring after renal ablation in particular on the first scans [60–63]. Major complication rates do not differ statistically between cryoablation (7.7%) and RF ablation (4.7%) [64]. If there is a concern for incomplete treatment, then a 3-month follow-up is recommended to prepare for second treatment as an early peripheral enhanced rim may be observed on CT or late MR sequences possibly in relation to inflammatory changes within the few weeks after treatment. Often located on the margins of an ablation, the presence of focal nodular enhancement remains the only validated imaging pattern of residual tumor tissue or recurrence. This requires scanning without, and then with the injection of contrast product.

On CT, the evaluation of the enhancement remains qualitative and quantitative. Any contrast enhancement of more than 15 HU should be considered significant [57, 65]. On MRI, due to the spontaneous high T1W signal intensity of the ablation zone, subtraction techniques must be used to detect or eliminate focal enhancement on margins. Quantitatively, an enhancement is considered significant on MRI if it exceeds 15% on the dynamic contrast-enhanced sequence [56]. After cryoablation, a more sustainable low T2W signal intensity rim can be observed [66]. Interruption of this rim might indicate a residual tumor. If complete treatment was performed, then a 12-month contrast-enhanced CT or MR is suggested including chest CT. Changes in the size of the treated site are observed. A volume increase is observed early and up to the first 2 months after the procedure, more particularly for small tumors less than 3 cm^3^ [67]. Then the volume of the scar may gradually decrease until 1 to 2 years. Involution of the ablation zone is more frequently observed after cryoablation. On CT and MRI, infiltration of peripheral fat is almost systematic, especially for exophytic tumors [67, 68]. A spontaneously dense and low T1W signal intensity peripheral halo is observed in nearly 75% of cases and appears during the first months and often persists [68]. Invagination of fat in the scar is rare and occurs later [67]. Renal ablation zone soft-tissue nodules from fat necrosis can appear long after ablation, enhance with contrast medium, mimic applicator tract or ablation zone tumor seeding, and may require biopsy for confirmation of benignity [69]. If necessary, biopsies should be performed 6 months after ablation and should not be systematic, based on a case-by-case discussion based on imaging findings [60, 69].

### Recommendations

- Follow-up imaging at 1 month after ablation to assess for complications and set baseline for future follow up by CT or MRI
- If there is a concern for incomplete treatment at 1 month, a 3-month follow-up is recommended to evaluate the need and prepare for a second treatment
- If complete treatment was performed, follow-up is recommended at 6 and 12 months
- Annual contrast-enhanced CT or MR are suggested up to 5 years
- If concern for recurrence, consider biopsy to confirm

## C. Follow-up after thermal ablation of lung tumors

### Computed tomography

CT remains the most often used technique for follow-up after thermal ablation for lung tumor with radiofrequency ablation (RFA), microwave ablation (MWA), or cryoablation (CRA). An increase in the size of the ablation zone is usually observed during the early post-ablation period because of inflammatory changes and hemorrhage. Before 6 months, the ablated area is supposed to exceed the size of the tumor before ablation [70]. Normally, the scar decreases moderately in size during follow-up but may remain almost stable after 6 months [71–73]. The definite scar is often equal to or bigger than the initial tumor. The baseline measurement of the ablation zone should be done 1 month after ablation on CT [70, 74–79]. RECIST evaluation after RFA or MWA in comparison to the baseline scan is not effective, due to the limitations of size measurements after ablation. After cryoablation, the downsizing of the ablation zone seems to be faster [80], so that size has a potential value during follow-up, measuring its surface [81], using RECIST [82], or WHO criteria [80]. In cases where the ablation zone remains stable, analysis of contrast enhancement is useful in the determination of the effectiveness of lung ablation [70, 75, 76, 78, 83]. When compared to pre-contrast CT, an increase of attenuation of at least 15 HU [82, 84–86] or 25 HU [74, 79] is described as suggestive of incomplete ablation. Apart from contrast uptake, any increase in size, nodular, irregular, or eccentric solid component appearing within or at the edge of the ablation zone, by comparing to the previous CT image, should be considered as a local recurrence [70, 72, 74, 76, 78, 85, 87, 88]. Early CT follow-up (until the 6th month) may demonstrate enlargement of hilar and mediastinal lymph nodes frequently (almost 60% of treated patients) with common reversibility as a result of the reactive change [89]. All these arguments support that CT should be performed with contrast injection. Recent technical improvements can be used to optimize the results of CT: dual-energy CT may help in the diagnosis of early recurrences after RF ablation [90] and perfusion CT performed pre- and postoperatively may be useful in the determination of adequate treatment after MWA [91].

#### FDG-PET-CT

PET-CT has been described as a useful method during follow-up after lung ablation [76, 92–96]. When PET is not regularly scheduled, it is mainly performed when local recurrence is suspected or systemic progression needs to be evaluated. In a prospective study, Bonichon et al. demonstrated the low specificity of PET-CT at the early period (3 months) after RF lung ablation, due to the high number of false- positive results [97], which was confirmed in another study evaluating follow-up of the ablation zone [92]. In a small cohort of 18 patients treated by RF and followed up for 24 months, SUV was described as a poor indicator of local recurrence since it may be equal to or greater than baseline SUV in almost half of the patients in the center of the ablation zone [98].

#### MRI

MR after lung ablation is only reported in a few recent preliminary studies, dedicated to early evaluation after RF [99] or MW [100]. A few years ago, another report described a short prospective series showing that early diffusion MR 3 days after RF may predict further local progression [101].

#### Recommendations

- Contrast-enhanced CT is the preferred modality
- PET-CT is recommended > 6 months after ablation, due to the high rate of false-positive results before 6 months
- PET-CT should be used when local progression is suspected at CT, when the whole-body evaluation is required, or to precisely target a suspected recurrence for biopsy.
- A contrast-enhanced CT is recommended before discharge of the patient to provide baseline measurements of the ablation zone and detect early complications
- A contrast-enhanced CT 1 month after ablation is recommended as baseline for further follow-up
- After 1 month, the ablation zone is expected to remain homogeneous without any significant enhancement, with gradual decrease in size and regular margins.
- After 6 months, the treated lesion may remain stable or slightly decrease in size after RFA or MWA. Shrinkage after cryoablation is usually more profound.
- Dual-energy CT, perfusion CT, and thoracic MR will possibly play a role in patient’s surveillance after lung ablation, but are still under investigation.

## Discussion

This paper presents recommendations based on expert opinion and evidence on follow-up after interventional radiological treatment for liver cancer, renal cancer, and lung cancer. Only limited evidence is available from small studies. The main aims of follow-up are identifying residual tumor/assessment of response, detecting recurrence and new tumors or metastases. CT, MRI, and (FDG-)PET-CT are the main modalities useful for follow-up. One of the main issues is that after oncological interventions standard RECIST 1.1 response assessment is not reliable and other response criteria should be used. Additionally, efforts should be made to evaluate new imaging techniques to further improve response assessment (e.g., exploration of DWI, DCE-MRI, perfusion CT).

All experts agreed that for all cancers discussed in this paper, follow-up at 1 month should be performed after the intervention to identify complications and possible residual tumor. After 1 month, the subsequent follow-up schedule is most strict for liver cancer compared to lung and renal cancer. For liver cancer, a follow-up schedule with imaging at 1, 3, 6, 9, 12 months and 6 monthly thereafter was proposed. Contrast-enhanced CT is mostly used in liver cancer in clinical practice but is not recommended by the panel as the primary modality. Even though CT is widely available, it can be performed quickly and is the modality that radiologists have most expertise with, MRI is the modality of choice for local evaluation in the liver mainly because its superiority to detect smaller lesions than CT. Furthermore, MRI does not require radiation or iodine contrast and in the specific issue of HCC, MRI is superior to ceCT when Lipiodol is used. When MRI is not easily available or there is a strong preference for CT in clinical practice, CT can be considered as well. In case of extrahepatic metastases that require monitoring with CT (e,g. for RECIST 1.1. evaluation), CT will be the modality of choice instead of MRI. During follow-up, it is not recommended to change modality as this can hamper comparability between two exams. For CT, texture analyses are currently under study and in due time this might be used in daily practice [102]. PET-CT can be considered in CRLM, but not for HCC. PET-CT is recommended as a problem solver and provides the advantage of high accuracy to detect extrahepatic metastases [103]. In the liver, hematoma and post-intervention changes can lead to false-positive DWI signal and FDG uptake in and around the treated area, and radiologists should be aware of these pitfalls when performing follow-up. For this reason, PET-CT is not recommended for the first 4–6 months after ablation for CRLM (unless it is performed within 48 h, but this is not available in most centers). After TARE, little evidence is available for follow-up, but PET-CT seems the most accurate early (4 weeks) modality for response evaluation for CRLM, as MRI can be false-positive due to inflammation early after TARE. DWI seems to be the best modality to identify local tumor progression. CT is the second recommended modality. RECIST 1.1 is not accurate for response assessment. Based on expert opinion the panel recommended Choi criteria as an alternative method for response assessment after TARE over RECIST 1.1, when using CT for follow-up. Even though, this still is suboptimal response assessment criteria (primarily developed for gastro-intestinal stromal tumor and low reproducibility), the panel did consider the Choi criteria superior to RECIST 1.1 [104–106].

For renal cancer, the follow-up schedule is less intensive but includes a 1, 3 (optional), 6 and 12-month follow-up in the first year, and after 1 year of follow-up, annual follow-up suffices. In renal cancer, both CT and MRI can be considered for follow-up and are considered equal in diagnostic performance during follow-up. PET-CT has no role as RCCs tend not to be FDG-avid.

For lung cancer, 1-month post-ablation contrast-enhanced CT is recommended and ceCT is the mainstay of follow-up. Just as for liver imaging, PET-CT should not be performed early after the intervention, given the risk for false-positive findings. PET-CT should be regarded as a problem solver and should be used when a recurrence is suspected.

A complicating factor in research on response assessment after the oncological intervention is the lack of a solid gold standard. Histopathology is rarely available and, therefore, the gold standard is constituted of (multiple) imaging modalities, which makes it very challenging to establish a good validation of imaging modalities. To improve the gold standard, a long follow-up interval is then needed, making these kinds of trials less appealing and cumbersome to execute. Efforts should be made to identify other validation techniques that are accurate and easy to use in clinical practice. The rapid development in interventional oncology treatments, but also in diagnostic techniques, stresses this need for a good gold standard and validation even more, in order to effectively incorporate new interventions into practice. Practically, when there is uncertainty in a clinical setting, a biopsy can always be considered in individual cases to provide more information (even though sampling error can be encountered due to difficulty to target the potential residual viable tissue in areas of necrosis after treatment).

The lack of high-quality prospective studies is an issue that needs attention. So far, small (sometimes) retrospective studies have been performed, without emphasis on follow-up modalities and schedules. Most studies in interventional radiology aim at evaluating success rates of procedures and follow-up is then usually performed with standard techniques, i.e., usually CT, which is known to have limitations, as discussed in this paper. However, from a diagnostic point of view, follow-up is important and should be evidence based and as accurate as possible. To improve this issue, it is advisable that interventional radiologists and diagnostic radiologist join efforts to improve research on response assessment after interventional radiology for cancer.

This consensus paper focuses on local treatment evaluation, but for follow-up metastases outside the treated organ should be considered as well. For CRLM and lung cancer, CT of the thorax and abdomen or PET-CT can be considered for distant staging. For renal cancer, CT is advisable (of the thorax and abdomen).

## Limitations

These recommendations have been constructed by use of evidence if available, but unfortunately, the quality and amount of evidence were limited. Therefore, the currently presented recommendations are partly based on expert opinion and consensus, which is the highest level of evidence available for some issues. We did not discuss who is responsible for follow-up: the interventional radiologist or the primary treating physician (e.g., surgeon, urologist, gastro-enterologist, or pulmonologist). It is important to prevent redundant follow-up, and therefore, interventional radiologists should make an effort to have a multidisciplinary follow-up protocol in their hospital in which it is clear who takes responsibility for follow-up. Last, thermal ablation of HCC was not discussed for which the LI-RADS treatment response algorithm is available [107].

## Conclusion

For liver cancer, renal cancer, and lung cancer, a follow-up schedule could be proposed after the oncologic intervention. PET-CT, CT, and MRI are the modalities of choice, but one should beware of false-positive signs of residual tumor or recurrence due to inflammation early after the intervention. Next to local evaluation, whole-body staging should be used as well, to identify lesions outside the targeted organ. The lack of high-quality evidence stresses the need for high-quality research on follow-up after radiological intervention in oncology and an accurate gold standard, especially with emerging new interventional and diagnostic techniques. For now, these recommendations can be used in clinical practice to guide the follow-up of patients that had a radiological intervention for liver cancer, lung cancer, and renal cancer.

## Data Availability

Evidence-based recommendations, no patient materials involved.

## Abbreviations

ADC: Apparent diffusion coefficient
AP: Arterial perfusion
ceCT: Contrast-enhanced computed tomography
CEUS: Contrast-enhanced ultrasound
CRLM: Colorectal liver metastases / metastatic colorectal cancer
DCE-MRI: Dynamic contrast-enhanced magnetic resonance imaging
DWI: Diffusion-weighted imaging
ECIO: European conference on interventional oncology
ESOI: European society of oncologic imaging
FDG-PET: Fluorodesoxyglucose positron emission tomography
GBCA: Gadolinium-based contrast agents
HCC: Hepatocellular carcinoma
HR: Hazard ratio
ICC: Intrahepatic cholangiocarcinoma
LTP: Local tumor progression
MDCT: Multi-detector computed tomography
mRECIST: Modified response evaluation criteria in solid tumors
MWA: Microwave ablation
PERCIST: PET Response Criteria in Solid Tumors
RCC: Renal cell cancer
RECIST: Response evaluation criteria in solid tumors
RFA: Radiofrequency ablation
SUVmax: Maximal standardized uptake value
TACE: Transarterial chemoembolization
TARE: Transarterial radioembolisation
Y90: Yttrium-90
